# Silent struggles: a machine learning approach for predicting suicidal ideation based on crisis symptoms and childhood trauma in Saudi adolescents

**DOI:** 10.1186/s40359-025-03830-6

**Published:** 2025-12-10

**Authors:** Mogeda El Sayed El Keshky, Radeah Mohammed Hamididin

**Affiliations:** 1 Department of Psychology, Faculty of Arts and Humanities, King Abdulaziz University, Jeddah, 42803 Kingdom of Saudi Arabia; 2 Department of Psychology, Faculty of Arts, Assiut University, Assiut, Arab Republic of Egypt

**Keywords:** Suicidal ideation, Adolescents, Saudi arabia, Emotional pain, Entrapment, Childhood trauma, Suicide crisis syndrome, Machine learning

## Abstract

**Background:**

Adolescent suicide is a significant public health concern, with rising rates globally and increasing psychological distress among youth. Despite its significance, limited research in Saudi Arabia has explored the combined impact of acute emotional crises and childhood trauma on suicidal ideation, particularly using machine learning approach.

**Objectives:**

This study aimed to identify key psychological and trauma-related predictors of suicidal ideation among Saudi adolescents. It specifically examined the role of Suicidal Crisis Syndrome symptoms and childhood maltreatment experiences using both correlational and machine learning approaches.

**Methods:**

A cross-sectional study was conducted among 583 Saudi adolescents aged 13–18 years, using convenience sampling. Participants completed a battery of validated self-report instruments: the Suicide Crisis Inventory, the Childhood Trauma Questionnaire–Short Form, and the Okasha Suicidality Scale. Descriptive statistics, bivariate correlations, and variable importance analysis via machine learning, in particular a random forest classifier, were used to identify the strongest predictors of suicidal ideation.

**Results:**

Emotional pain, entrapment, and panic dissociation were the most significant predictors of suicidal ideation. Childhood trauma variables—particularly emotional abuse, sexual abuse, and emotional neglect—also showed strong positive correlations with suicidal ideation. Adolescents from divorced families and those in high school reported significantly higher suicidal ideation scores. The random forest model confirmed emotional pain and entrapment as top predictors.

**Conclusions:**

Suicidal ideation among Saudi adolescents is strongly influenced by both acute emotional crisis symptoms and histories of childhood trauma. These findings highlight the need for early screening of suicidal crisis markers and trauma-informed mental health interventions in school and clinical settings. Culturally sensitive strategies that integrate emotional, developmental, and family-based factors are essential for effective suicide prevention among adolescents.

## Background

Adolescent suicide is a critical global public health challenge, ranking among the leading causes of death in this age group [1]. The developmental period of adolescence is marked by profound emotional, cognitive, and social changes that can increase vulnerability to mental health difficulties, including suicidal ideation and behavior. More specifically, according to the World Health Organization, suicide is the fourth leading cause of death among individuals aged 15–29 years, with an estimated rate ranging between 3 and 8 per 100,000, depending on the region [1]. In the Arab world, although comprehensive data remain limited due to underreporting and cultural stigma, studies suggest a rising prevalence of suicide among youth, with rates estimated between 4.3% and 11.4% [2]. In Saudi Arabia, adolescent suicide rates are relatively lower, estimated between 1.2 and 3 per 100,000, yet recent surveys have reported that up to 10–20% of adolescents experience suicidal thoughts, particularly females and those with untreated psychological distress [3]. While the etiology of suicidality is known to be multifactorial, involving psychological, environmental, and interpersonal risk factors, the precise mechanisms through which these influences interact remain a subject of ongoing investigation.

In recent years, increasing scholarly interest has focused on immediate affective and cognitive states as critical triggers for suicidal crises. Traditional risk models, which rely heavily on static or demographic variables (e.g., prior attempts, diagnosis), have shown limited predictive efficacy for imminent risk [4]. Suicidal Crisis Syndrome (SCS) is a theoretically grounded model describing the acute affective and cognitive state that precedes suicidal behavior. SCS encompasses five interrelated components—emotional pain, perceived entrapment, panic-dissociative symptoms, ruminative flooding, and fear of dying—and has been shown to predict near-term suicidal thoughts and behaviors more accurately than traditional risk factors [5]. Multiple studies have demonstrated its utility across clinical and community samples: for example, the Suicide Crisis Inventory (SCI) has shown strong internal consistency and predictive validity for short-term suicidal behavior, and recent systematic reviews confirm the syndrome’s diagnostic relevance [6]. Evidence from multinational samples also suggests that SCS indicators are present across culturally diverse populations, supporting the generalizability of the framework [7]. Furthermore, SCS-based tools have improved clinical decisions in emergency settings, highlighting their practical value in assessing imminent risk [8]. This growing body of research positions SCS as a useful model for examining dynamic suicide risk factors among adolescents, whose emotional reactivity and developmental transitions may heighten vulnerability to acute crisis states.

Parallel to the emotional predictors of suicidality, exposure to childhood trauma has consistently emerged as a potent distal risk factor. Experiences such as emotional abuse, physical neglect, and sexual abuse have been shown to have enduring effects on emotional regulation, cognitive functioning, and self-concept, each of which may contribute to increased suicide risk during adolescence [9, 10]. Recent meta-analytic evidence strongly supports the role of adverse childhood experiences (ACEs) in suicidal behaviors across diverse clinical populations. For instance, a meta-analysis of 41 studies across 17 countries found that individuals with affective disorders exposed to ACEs had nearly double the odds of engaging in suicidal behavior [11]. Moreover, a large longitudinal cohort study from the U.S. found that emotional, physical, and sexual abuse before age 18 predicted similar increases in adult suicidal ideation—between 30% and 50% of that effect was shown to be mediated via psychological distress, perceived powerlessness, and social rejection [12]. Importantly, recent research specific to non-Western and Middle Eastern contexts supports these global trends. In a cohort of Saudi adolescents and young adults aged 17–21, over 85% reported at least one ACE. Those exposures significantly predicted poorer subjective well‑being and higher frequencies of health complaints, with sense of mastery partially mediating these effects [13]. However, research that jointly considers both proximal psychological states and historical trauma exposures in adolescent populations, particularly in non-Western contexts such as Saudi Arabia, remains sparse.

The present study seeks to bridge a critical gap in the literature by examining the relative contributions of suicidal crisis symptoms and childhood trauma to suicidal ideation among Saudi adolescents. A major strength of this study lies in its use of machine learning, in particular a random forest algorithm, to assess variable importance. Unlike traditional statistical techniques, machine learning models can capture complex, non-linear relationships and interactions among predictors without relying on strict parametric assumptions [14, 15]. This data-driven approach enhances the robustness of the findings by prioritizing features based on their predictive power, thereby offering more nuanced insights into the determinants of suicidal ideation. Such methodology is especially well-suited for high-dimensional psychological data, where multiple interrelated factors often co-occur.

Findings from this study are intended to inform culturally sensitive mental health interventions and enhance suicide prevention strategies targeted at adolescents in Saudi Arabia. By illuminating both the emotional states that accompany suicidal crises and the longer-term impact of childhood trauma, this research offers a nuanced understanding of suicidality that may be applicable in both clinical and educational settings. To our knowledge, this is the first study in the Arab region to simultaneously examine proximal crisis-state markers (SCS symptoms) and distal childhood trauma indicators using a machine learning framework. Previous regional studies have primarily documented prevalence patterns or simple correlational associations. The present work extends this literature by integrating theoretically grounded crisis constructs with high-dimensional data modeling, thereby offering a more nuanced and culturally specific understanding of suicide risk among Saudi adolescents.

### Aim of the study

This study aimed to identify the key psychological, emotional, and trauma-related predictors of suicidal ideation among Saudi adolescents. Specifically, it sought to answer the following research questions:

1. Which Suicidal Crisis Syndrome symptoms are most strongly associated with suicidal ideation?
2. How do different forms of childhood trauma relate to suicidal ideation?
3. Which psychosocial variables emerge as the strongest predictors when examined through a machine learning model?

## Methods

### Study design and sampling

A cross-sectional quantitative design was used alongside convenience sampling to maximize participant diversity and reach.

### Participants

This research targeted adolescent populations in Saudi Arabia. Recruitment was facilitated through schools, and participants were encouraged to share the questionnaire with their peers via social media platforms such as Facebook, WhatsApp, and Twitter. Although 611 adolescents initially responded, only 583 completed the questionnaire without significant missing data and thus were included in the final analysis. Participants were fully informed about the study’s objectives and intended outcomes, and informed consent was obtained prior to participation. Ethical approval for the study was granted by King AbdulAziz University in Saudi Arabia.

### Instruments

Validated psychometric tools, including the Suicide Crisis Inventory [5], the Childhood Trauma Questionnaire [18], and the Okasha Suicidality Scale [17], were employed to ensure reliable measurement of key variables. In addition, the survey incorporated sociodemographic items.

#### Translation & pilot study

All instruments were translated using a forward–backward translation procedure by bilingual mental health professionals. Minor discrepancies were resolved through consensus. The Arabic versions of the scales were adapted following recommended translation guidelines. Subsequently, a pilot study was conducted with a small sample of 39 participants to assess clarity, cultural relevance, and comprehension of the items. Feedback from the pilot led to minor wording adjustments to ensure that the questions were easily understood and contextually appropriate.

#### The suicide crisis inventory

[5] is a 49-item self-report tool designed to assess suicidal crisis syndrome by examining individuals’ psychological state in the days immediately preceding the assessment. Items are rated on a five-point Likert scale, ranging from 0 (not at all) to 4 (extremely). The scale measures key dimensions of the syndrome, such as entrapment, ruminative flooding, fear of death, panic dissociation, and emotional distress. A total score can be made, ranging between 0 and 196, with a cut-off score of 102. Previous research demonstrated strong psychometric validity for this tool [16]. In the present study, the total score was analyzed, showing high internal consistency (α = 0.93 for entrapment, α = 0.86 for panic dissociation, α = 0.84 for ruminative flooding, α = for fear of dying 0.76, and α = 0.89 for emotional pain).

#### The okasha suicidality scale

[17] consists of five items assessing suicidal ideation and behavior. The first four items evaluate the frequency of suicidal thoughts using a four-point scale (0 = never to 3 = often), while the fifth item measures the frequency of suicide attempts, with responses ranging from 0 (no attempts) to 3 (three or more attempts). In this study, the scale demonstrated excellent internal reliability (α = 0.88). The Okasha Suicidality Scale total score (range 0–15) was used as a continuous index of suicidal ideation. For machine learning classification, participants scoring ≥ 5 were categorized as “elevated ideation,” consistent with prior regional research.

#### The childhood trauma questionnaire–short form

[18] includes 28 items that assess five types of childhood maltreatment: emotional abuse, physical abuse, sexual abuse, emotional neglect, and physical neglect. Each subscale contains five items rated on a five-point Likert scale from 1 (never true) to 5 (very often true). Thus, total scores for each dimension range from 5 to 25. Additionally, three items serve to detect socially desirable responding. The cut-off scores are as follows: ≥ 13 for Emotional Abuse; ≥ 10 for Physical Abuse; ≥ 8 for Sexual Abuse; ≥ 15 for Emotional Neglect; ≥ 10 for Physical Neglect. Reliability analysis showed good internal consistency across the subscales: physical abuse (α = 0.83), emotional abuse (α = 0.77), sexual abuse (α = 0.75), physical neglect (α = 0.76), and emotional neglect (α = 0.87).

### Data analysis

All data preprocessing and statistical analyses were conducted using RStudio [19], a powerful and flexible software environment for data science. To predict suicidal ideation, we implemented a random forest classifier using the *randomForest* package in R. Random forest is an ensemble learning technique that constructs a multitude of decision trees during training and outputs the class that represents the mode of each individual tree’s predictions [14]. This approach is particularly advantageous for its ability to handle both categorical and continuous variables, as well as for its robustness in dealing with noisy or imbalanced data.

In this study, the random forest algorithm was employed due to its high classification accuracy, resistance to overfitting, and capacity to model complex interactions between variables without requiring extensive data preprocessing or assumptions about variable distributions. Specifically, it is well-suited for binary classification tasks such as the identification of individuals with elevated levels of suicidal ideation. One key strength of this method is its ability to rank predictor importance, providing valuable insights into which factors most strongly influence model predictions. To evaluate model performance, the dataset was randomly partitioned into two subsets: 70% of the data was allocated to training the model, while the remaining 30% was used for testing and validation. This train-test split allowed for a more reliable assessment of model generalizability to unseen data. By leveraging the ensemble structure and feature selection mechanism of random forest, the analysis was able to identify the most influential psychosocial predictors of mental health outcomes with a high degree of reliability.

To clarify model specification, we included age and gender as control variables in the random forest model, and these were entered alongside all psychological and trauma-related predictors. No cases had missing values on model variables; therefore, no imputation procedures were required. Random forests are inherently robust to predictors measured on different scales, so no normalization or standardization was applied. Class balance was examined prior to modeling: 36% of participants met criteria for elevated suicidal ideation, indicating only a mild imbalance; nonetheless, the algorithm’s out-of-bag sampling and bootstrapping procedures helped maintain balanced performance across classes.

Before modeling, Multicollinearity was assessed using variance inflation factors (VIF < 4 for all SCS subscales), and no variables were removed. Random forests are tolerant to correlated predictors, so no exclusion was required. Suicidal ideation was treated as continuous for correlational analyses and dichotomized for classification purposes. Ten-fold cross-validation was conducted to complement the 70/30 train-test split; cross-validated accuracy = 0.89, AUC = 0.91.

## Results

### Sociodemographic factors

Table 1 presents the sociodemographic profile of the participants. Females constituted 52.8% of the sample, with a mean participant age of 15.8 years (SD = 1.79), ranging from 13 to 18 years. Approximately 52.8% of respondents were enrolled in high school, while 47.2% attended intermediate school at the time of the study. Regarding paternal education, 18.9% of participants reported that their fathers had not completed high school, 14.6% had fathers with a high school diploma, 50.9% had fathers with a bachelor’s degree, and 15.6% had fathers with a postgraduate qualification. Maternal education levels showed that 16% of mothers had less than a high school education, 25.6% completed high school, 44% held a college degree, and 14.4% had a postgraduate degree.

In terms of family structure, 74.4% of participants lived with both parents, 11.3% were raised by a single parent, 12.6% had divorced parents, and 1.7% had lost one or both parents. Household income varied, with 19.4% earning less than 5,000 Saudi Riyals (SR) monthly, 17.5% earning between 5,000 and 10,000 SR, 23.5% between 10,000 and 15,000 SR, 20.2% between 15,000 and 20,000 SR, and 19.4% earning more than 20,000 SR per month.

Analysis revealed significant differences in suicidal ideation based on parental marital status and education level, with higher levels of ideation observed among high school students and those with divorced parents (*p* < 0.001).

**Table 1:** Sociodemographic characteristics of the sample

					Suicidal ideation	
Variables			N		Mean (SD)	*p*
Gender						*0.420*
	Male		275	47.2	4.7 (2.8)	
	Female		308	52.8	4.9 (3.6)	
Education level						*< 0.001*
	Intermediate		275	47.2	4.40 (3.17)	
	High school		308	52.8	5.36 (3.32)	
Marital status of parents						*< 0.001*
	Live together		434	74.4	4.50 (3.07)	
	Single parent		66	11.3	5.40 (3.25)	
	Divorced		73	12.6	6.84 (3.76)	
	Parents are dead		10	1.7	5.10 (3.72)	
Father’s education						*0.752*
	Less than high school		110	18.9	5.37 (4.03)	
	High school		85	14.6	3.96 (3.42)	
	Bachelor		297	50.9	4.84 (2.68)	
	Postgraduate		91	15.6	5.41 (3.77)	
Mother’s education						*0.404*
	Less than high school		93	16	5.17 (3.43)	
	High school		149	25.6	5.37 (3.75)	
	Bachelor		257	44	4.31 (2.68)	
	Postgraduate		84	14.4	5.59 (3.66)	
Income						*0.357*
	< 5000 SR		113	19.4	4.84 (3.75)	
	5000–10,000 SR		102	17.5	5.32 (4.33)	
	10,000–15,000 SR		137	23.5	5.28 (2.82)	
	15,000–20,000 SR		170	29.2	4.28 (2.07)	
	> 20,000 SR		61	10.4	5.22 (3.83)	

### Descriptive statistics and correlation analysis

Table2 displays the descriptive statistics and the correlation matrix for the study variables. The average score for suicidal ideation was 4.90 (SD = 3.28). Mean scores for other psychological and trauma-related variables were as follows: entrapment (M = 31.6, SD = 11.4), panic dissociation (M = 20.2, SD = 8.14), ruminative flooding (M = 17.9, SD= 5.8), fear of dying (M = 7.43, SD = 2.49), emotional pain (M = 9.54, SD = 4.04), physical abuse (M = 9.76, SD = 3.19), emotional abuse (M = 13.16, SD = 2.9), sexual abuse (M = 13.49, SD = 3.5), physical neglect (M = 5.73, SD = 3.11), and emotional neglect (M = 17.7, SD = 2.88). Suicidal ideation was significantly and positively associated with several variables. It showed a weak but significant correlation with age (r = 0.23, p < .01). Stronger positive correlations were observed between suicidal ideation and entrapment (r = 0.51, p < .001, 95% CI: 0.45–0.57), panic dissociation (r = 0.41, p < .001, CI: 0.34–0.48), ruminative flooding (r = 0.41, p < .001, CI: 0.32–0.49), fear of dying (r = 0.35, p < .001, 95% CI: 0.28–0.42), and emotional pain (r = 0.52, p < .001, 95% CI: 0.46–0.58). Additionally, suicidal ideation was significantly correlated with experiences of physical abuse (r = 0.24, p < .001, 95% CI: 0.22–0.30), emotional abuse (r = 0.28, p < .001, 95% CI: 0.21–0.36), sexual abuse (r = 0.33, p < .001, 95% CI: 0.25–0.40), physical neglect (r = 0.39, p < .001, 95% CI: 0.35–0.43), and emotional neglect (r = 0.29, p < .001, 95% CI: 0.21–0.36).

**Table 2:** Descriptive statistics and correlation matrix of the variables

Variable	Mean (SD)	1	2	3	4	5	6	7	8	9	10	11	12
1. Age	15.8 (1.79)	1											
2. Suicidal ideation	4.90 (3.28)	0.23**	1										
3. Entrapment	31.06 (11.4)	−0.06	0.51***	1									
4. Panic dissociation	20.2 (8.14)	−0.14	0.41***	0.70***	1								
5. Ruminative flooding	17.9 (5.8)	−0.01	0.41***	0.72***	0.64***	1							
6. Fear of dying	7.43 (2.49)	−0.08	0.35***	0.49***	0.39***	0.40***	1						
7. Emotional pain	9.54 (4.04)	−0.08	0.52***	0.76***	0.79***	0.70***	0.35***	1					
8. Physical abuse	9.76 (3.19)	0.14*	0.24***	0.34***	0.32***	0.18**	0.29***	0.22***	1				
9. Emotional abuse	13.16 (2.9)	0.12	0.28***	0.28***	0.35***	0.33***	0.22***	0.28***	0.56***	1			
10. Sexual abuse	13.49 (3.5)	−0.11	0.33***	0.15*	0.23**	0.19***	0.33***	0.20***	0.23**	0.20**	1		
11. Physical neglect	5.73 (3.11)	0.22**	0.39***	0.17*	0.18*	0.20***	0.28***	0.18*	0.48***	0.54***	0.54***	1	
12. Emotional neglect	17.7 (2.88)	−0.02	0.29***	0.24***	0.26***	0.29***	0.32***	0.21***	0.45***	0.52***	0.52***	0.32***	1

* *p* < 0.05; **p < 0.01 *** *p* < 0.001

### Machine learning model performance evaluation

**Fig. 1:**
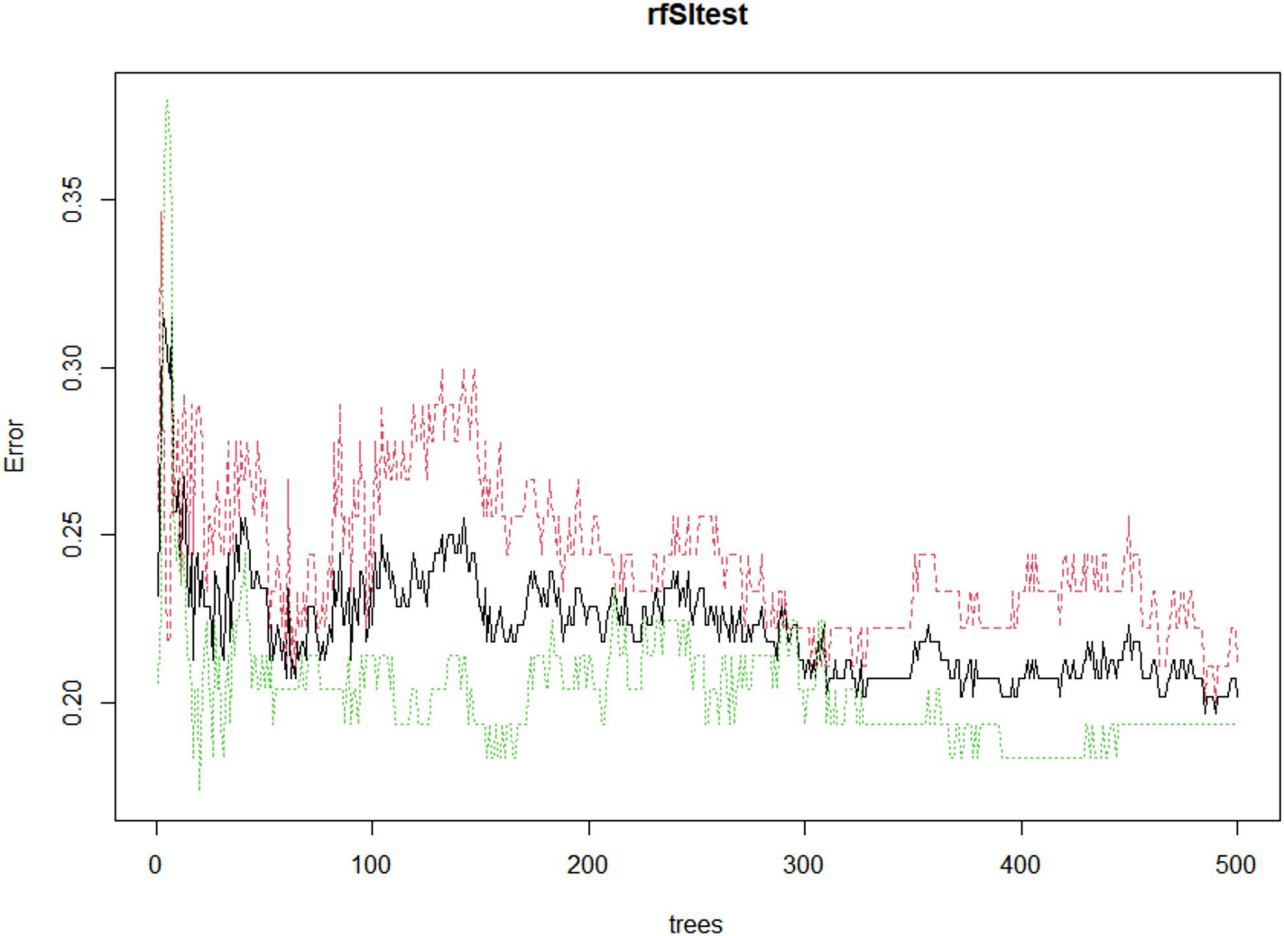
Error rate of random forest

Figure 1 presents the out-of-bag (OOB) error rate trajectory across 500 trees constructed by the random forest model. The horizontal axis denotes the number of trees, while the vertical axis reflects the corresponding error rates. The black solid line illustrates the overall OOB error, whereas the green and red dashed lines represent class-specific error rates for each outcome category.

As the number of trees increases, a noticeable reduction in error rates is observed, particularly during the early stages of tree growth. The error rates progressively stabilize beyond approximately 300 trees, indicating that additional trees contribute minimal improvement to model performance. By the 500th tree, the overall OOB error rate converges to a value below 0.20, suggesting strong classification capability. The relatively low and stable class-specific error rates further confirm the model’s robustness and consistency across different outcome classes. This graphical output supports the conclusion that the selected model parameters—500 trees and 4 variables per split—yield a well-calibrated and high-performing model without evidence of overfitting.

### Model prediction

The dataset was partitioned into two subsets: 70% allocated for training and 30% for testing on participant data. To evaluate the predictive performance of the random forest model, several metrics were employed, including out-of-bag (OOB) error estimates, confusion matrices, specificity, and sensitivity. For the training dataset, the model yielded an OOB error rate of 10.52%, corresponding to an accuracy of 89.48%, using 500 decision trees and four variables at each split. The confusion matrix further validated the model’s effectiveness, demonstrating an overall accuracy of 0.917 (95% CI: 0.90–0.965), with a specificity of 0.932 and sensitivity of 0.94, indicating highly accurate classification.

**Fig. 2:**
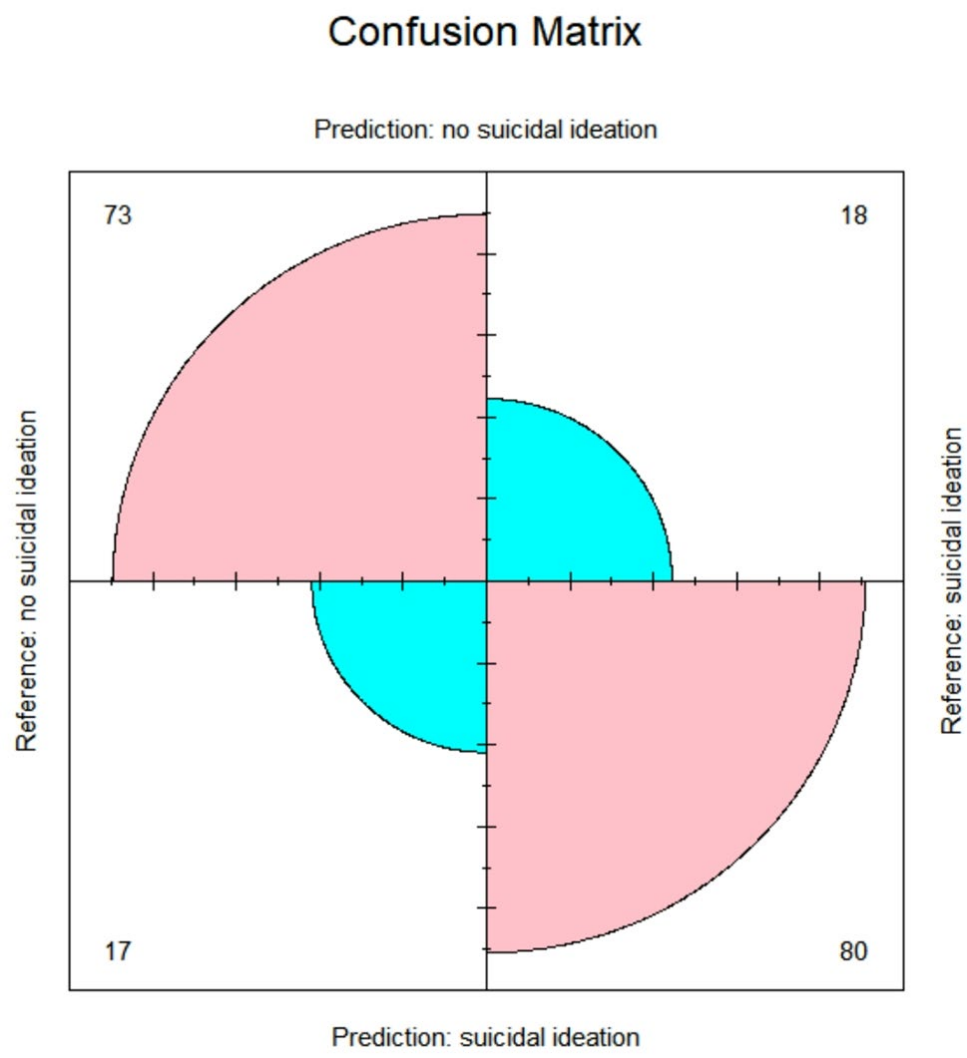
Confusion matrix

Given this robust performance, the model was subsequently applied to the test dataset using the same parameters. The test results showed an accuracy of 0.907 (95% CI: 0.88–0.926), with a specificity of 0.91 and sensitivity of 0.89, reflecting strong predictive capability. The model achieved an AUC of 0.91, precision of 0.88, and recall of 0.90 on the test set, confirming strong discriminative performance. As the model performed well on both datasets, no additional tuning was deemed necessary. Figure 2 presents the corresponding confusion matrix. Feature importance values are also presented numerically in Supplementary Table 1.

### Variable importance and SHAP values

**Fig. 3:**
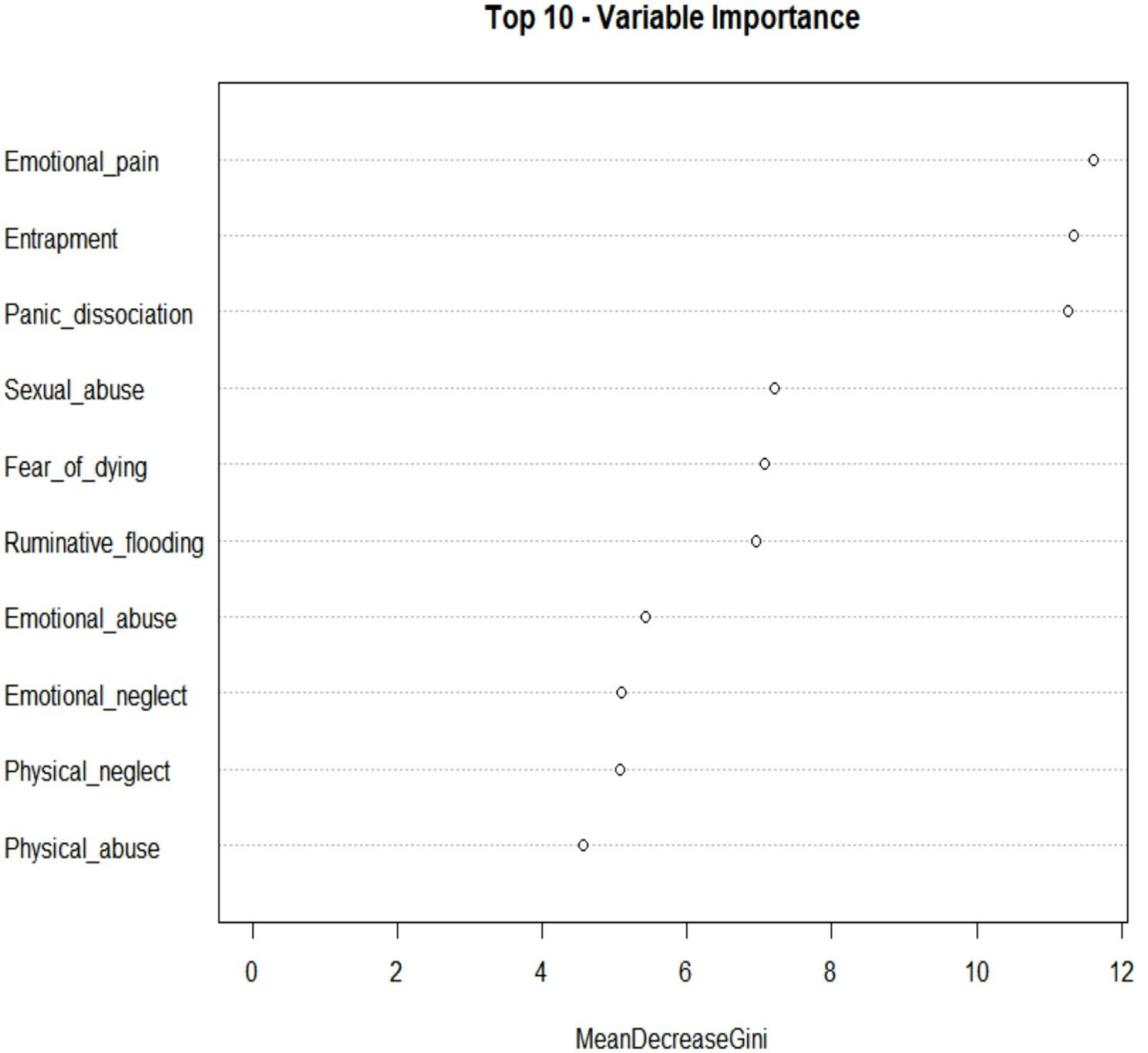
Variable importance

**Fig. 4:**
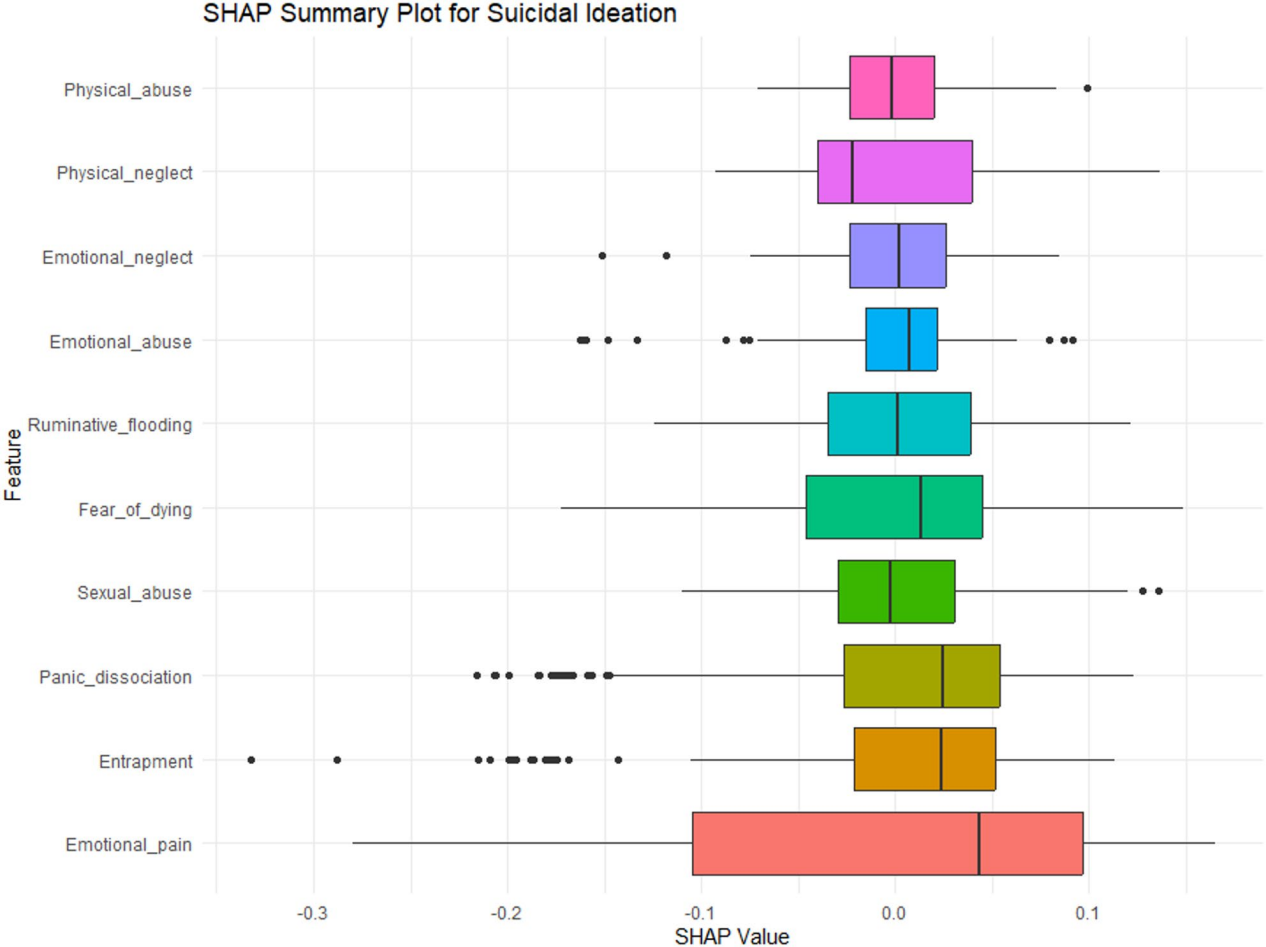
SHAP values plot

Figure 3 presents the top ten predictors of suicidal ideation based on their importance in the random forest model, as measured by the Mean Decrease in Gini index. Among all variables, emotional pain emerged as the most influential predictor, followed closely by entrapment and panic dissociation. Sexual abuse, fear of dying, and ruminative flooding also demonstrated notable importance. Additional variables contributing to the model included emotional abuse, emotional neglect, physical neglect, and physical abuse, though their relative influence was comparatively lower. These findings are supported by the SHAP (SHapley Additive exPlanations) values plotted in Fig. 4. The mean absolute SHAP values are displayed in Table 3. Among all variables, emotional pain emerged as the most influential factor, exhibiting the largest and most consistently positive SHAP values. This finding highlights the salience of acute affective distress in driving suicidal thoughts, consistent with the theoretical framework of the Suicidal Crisis Syndrome. Entrapment, another core SCS symptom, also demonstrated a substantial impact, reinforcing the notion that feeling unable to escape one’s emotional state and, perhaps, distressing situation is a key precipitant of suicidal ideation. These findings highlight the prominence of acute emotional and psychological distress variables over historical trauma in predicting suicidal ideation.

**Table 3:** Mean absolute SHAP values

Rank	Predictor Variable	Mean Absolute SHAP Value	Interpretation (Higher Value = Greater Contribution)
1	Emotional pain	0.142	Strongest predictor; major driver of elevated SI
2	Entrapment	0.086	High perceived inescapability strongly increases SI
3	Panic dissociation	0.074	Dissociative panic symptoms substantially influence SI
4	Sexual abuse	0.072	Most influential trauma variable
5	Fear of dying	0.063	Contributes moderately to acute crisis states
6	Ruminative flooding	0.050	Repetitive intrusive thoughts elevate SI
7	Emotional abuse	0.044	Emotional maltreatment exerts a notable effect
8	Emotional neglect	0.036	Chronic emotional deprivation influences vulnerability
9	Physical neglect	0.034	Increases SI risk but less strongly than emotional factors
10	Physical abuse	0.033	Smallest but still meaningful contribution

## Discussion

The present study investigated the psychological and trauma-related predictors of suicidal ideation among Saudi adolescents, using validated assessment tools and a combination of traditional statistics and machine learning–based variable importance analysis. The findings underscore the significant role of both acute emotional disturbances and childhood trauma in shaping suicidal thoughts during adolescence. To our knowledge, this is the first study in the Arab region to use machine learning to examine both proximal and distal predictors of adolescent suicidal ideation.

Consistent with the Suicidal Crisis Syndrome framework proposed by [5], several emotional and cognitive symptoms—including emotional pain, entrapment, panic dissociation, ruminative flooding, and fear of dying—were strongly correlated with suicidal ideation. Notably, emotional pain emerged as the most important predictor, followed closely by entrapment and panic dissociation, according to the Mean Decrease Gini index in the random forest analysis. These findings align with prior studies suggesting that intense emotional suffering and a perceived lack of escape from psychological distress are among the most immediate precursors to suicidal thinking and behavior [6, 20]. This aligns with the Emotional pain and social Disconnect (END) neurobiological model, which identifies aberrant activation in emotional pain circuits (e.g., amygdala, hippocampus) among suicidal youth [21]. Likewise, systematic reviews confirm that feelings of defeat and entrapment are central psychological drivers of suicidal ideation, especially in adolescents [22].

This emphasis on dynamic emotional states reflects a growing consensus in suicidology that acute psychological processes may better predict near-term suicide risk than static risk factors such as demographic characteristics [23]. Emotional pain, in particular, is thought to represent an internal state of unbearable psychological suffering, often accompanied by feelings of hopelessness, defeat, or existential dread. Entrapment—the perception of being stuck in an intolerable situation with no means of escape—further amplifies this risk, as it may impair cognitive flexibility and promote suicidal ideation as a perceived solution [24].

In addition to emotional states, this study found robust associations between childhood trauma and suicidal ideation. Emotional abuse, emotional neglect, sexual abuse, and physical neglect all demonstrated significant correlations with suicidal ideation. Among these, sexual abuse and emotional abuse were particularly influential according to the variable importance analysis. These results reinforce a substantial body of literature linking adverse childhood experiences (ACEs) to long-term mental health difficulties, including depression, anxiety, and suicidal behavior [9, 10]. Trauma exposure, particularly sexual and emotional abuse, was strongly correlated with suicidal ideation. These variables also ranked prominently in the variable importance analysis, indicating they significantly contribute beyond emotional crisis symptoms. These findings are consistent with large-scale epidemiological evidence showing that cumulative childhood trauma markedly increases suicidality risk among youth [25]. In a meta-analysis of 21 studies involving individuals with schizophrenia-spectrum disorders, ACE exposure was associated with a significantly elevated risk of suicidal ideation and attempts [26]. These findings support the conceptualization of trauma not just as a background risk but as a core component in suicide risk models. In the Arab world, research indicates that childhood adversities significantly predict suicidal thoughts and behaviors, notwithstanding perhaps deceptively low overall suicide rates due to under-reporting and cultural stigma [27].

The findings also reveal important sociodemographic patterns. Adolescents enrolled in high school and those from divorced families reported higher levels of suicidal ideation. This may reflect increased academic pressures and developmental stress during late adolescence, as well as the destabilizing effects of family disruption on adolescent emotional security. Previous research in Middle Eastern contexts has emphasized the protective role of family cohesion and parental support in adolescent well-being [28], suggesting that divorce may significantly disrupt these protective factors. Regional evidence from network analysis studies in Saudi Arabia underscores the protective role of family cohesion and positive parenting, directly opposing risk factors such as psychache and bullying victimization [29]. Moreover, the positive correlation between age and suicidal ideation in this study suggests that older adolescents may face more severe psychological burdens than their younger counterparts. This trend may reflect increased awareness of life stressors, identity confusion, and future uncertainty during the transition to adulthood [30].

Together, these results support an integrated model of suicidality that incorporates both proximal emotional factors and distal developmental experiences. Early trauma may heighten emotional dysregulation, increasing vulnerability to crisis states like entrapment. From a clinical perspective, this underscores the importance of early screening for both current emotional crises and historical trauma. Mental health professionals working with adolescents, particularly in school settings, should be equipped to assess not only depression and suicidal thoughts, but also experiences of emotional pain, cognitive entrapment, and dissociative symptoms that may signal acute risk.

The integration of SCS constructs with childhood trauma within a machine learning model represents a novel contribution to suicidology in the Arab region, where studies seldom combine dynamic crisis markers with distal developmental risk factors.

We found that adolescents with postgraduate-educated parents exhibited higher suicidal ideation although the difference was not statistically significant. This may reflect heightened academic expectations, intense performance pressure, and more achievement-oriented parenting styles commonly documented in highly educated families. Such environments, while supportive in many ways, may inadvertently increase internalized pressure to excel, contributing to emotional distress and feelings of inadequacy.

In the Saudi context, cultural and religious norms strongly discourage the open expression of suicidal thoughts, several studies in the region have noted that certain socially shared beliefs about mental illness—such as the perception that psychological difficulties may reflect personal weakness or a lack of spiritual resilience—can reduce adolescents’ willingness to disclose their emotional struggles or seek early support [31]. As mental-health and suicide-related stigma persists, many adolescents may feel reluctant to openly discuss their distress or access available support services, even when they are in significant need [32]. These cultural factors underscore the importance of sensitive, non-judgmental screening approaches.

### Limitations

Despite the strengths of this study, several limitations warrant attention. First, the cross-sectional design precludes causal inference. Longitudinal studies are needed to examine how suicidal crisis symptoms and trauma exposure interact over time. Second, although convenience sampling allowed access to a large and diverse sample, it limits the generalizability of the findings. This may have introduced self-selection bias, potentially oversampling adolescents with greater digital literacy or openness to psychological surveys. Future studies should aim for more representative sampling methods across different regions of Saudi Arabia. Third, the reliance on self-report measures may introduce social desirability or recall bias, particularly in reporting sensitive experiences such as abuse or suicidal thoughts. Incorporating clinical interviews or multi-informant data could improve the robustness of future research. Fourth, SCS symptoms and suicidal ideation were measured concurrently, so temporal precedence can’t be established. Finally, cultural factors specific to Saudi Arabia, such as stigma surrounding mental health and limited public discourse on suicide, may influence the accuracy of self-disclosures. Further qualitative studies could help unpack the cultural nuances that shape adolescents’ understanding and expression of psychological distress.

## Conclusions

This study contributes to the growing body of evidence on adolescent suicidality by demonstrating that both acute psychological crises and early-life trauma significantly predict suicidal ideation among Saudi youth. The findings affirm that emotional pain, entrapment, and panic dissociation—core components of the Suicidal Crisis Syndrome—are among the most powerful predictors of suicidal thoughts. In parallel, experiences of emotional and sexual abuse, as well as emotional and physical neglect, were shown to exert substantial long-term influence. These results underscore the need for an integrated model of suicide risk that addresses both proximal emotional states and distal trauma-related experiences. Moreover, the sociocultural and developmental context—such as family structure and academic stage—also plays a critical role in shaping vulnerability. Taken together, this multidimensional understanding of risk can inform more targeted, effective suicide prevention efforts in both clinical and educational settings. Prioritizing early detection, trauma recovery, emotional regulation, and digital innovation may contribute to culturally adaptive suicide prevention strategies in Saudi Arabia and broader Arab societies.

.

### Implications

These findings hold several practical implications. First, suicide prevention efforts in Saudi Arabia should adopt trauma-informed approaches, recognizing the long-lasting impact of childhood abuse and neglect on mental health. Second, given the prominence of emotional pain and crisis-related symptoms, interventions such as crisis stabilization, emotion regulation training, and brief psychotherapeutic interventions targeting suicidal thinking may be particularly effective. School counselors and mental health practitioners must be trained to recognize signs of SCS, not just depressive symptoms. In terms of policy, the results highlight the need for mental health infrastructure within schools, especially at the high school level. Programs that promote emotional resilience, family support, and early trauma screening could serve as protective mechanisms against the development of suicidal ideation. Finally, digital or AI-based screening tools could be used as a potential application of the machine learning findings (e.g., early detection in school or primary care settings).

## Data Availability

The data set used and/or analyzed during the current study available from the author on request.
